# Separation of the inelastic and elastic scattering in time-of-flight mode on the pinhole small-angle neutron scattering diffractometer K­WS-2

**DOI:** 10.1107/S1600576721006610

**Published:** 2021-07-30

**Authors:** Livia Balacescu, Georg Brandl, Aurel Radulescu

**Affiliations:** a Jülich Centre for Neutron Science (JCNS) at Heinz Maier-Leibnitz Zentrum (MLZ), Forschungszentrum Jülich GmbH, Lichtenbergstrasse 1, Garching, 85747, Germany; b Physikalisches Institut (IA), Rheinisch-Westfälische Technische Hochschule (RWTH), Otto-Blumenthal Strasse, Aachen, 52074, Germany

**Keywords:** TOF-SANS, incoherent neutron scattering, inelastic neutron scattering, hydro­carbon systems

## Abstract

To study and control the incoherent inelastic background in small-angle neutron scattering, which makes a significant contribution to the detected scattering from hydrocarbon systems, the KWS-2 small-angle neutron scattering diffractometer operated by the Jülich Centre for Neutron Science (JCNS) at Heinz-Maier Leibnitz Zentrum (MLZ), Garching, Germany, was equipped with a secondary single-disc chopper that is placed in front of the sample stage. This makes it possible to record in time-of-flight mode the scattered neutrons in the high-*Q* regime of the instrument (*i.e.* short incoming wavelengths and detection distances) and to discard the inelastic component from the measured data. Examples of measurements on different materials routinely used as standard samples, sample containers and solvents in the experiments at KWS-2 are presented. When only the elastic region of the spectrum is used in the data-reduction procedure, a decrease of up to two times in the incoherent background of the experimentally measured scattering cross section may be obtained. The proof of principle is demonstrated on a solution of bovine serum albumin in D_2_O.

## Abstract 2

A new chopper has been installed at the sample position in front of the sample stage at the KWS-2 small-angle neutron-scattering diffractometer of the Jülich Centre for Neutron Science. The pulsed beam and the time-of-flight data acquisition enable the separation of elastic and inelastic scattering from hydrogenous samples.

## 1. Introduction   

In a small-angle neutron scattering (SANS) experiment one measures the coherent scattering, which bears structural information about the sample and is predominantly elastic, and the *Q*-independent incoherent scattering. In the case of soft-matter and biophysical-related samples, which to a large extent are hydro­carbon systems, the incoherent ‘background’ originates mostly from scattering on hydrogen and deuterium nuclei in the sample. This incoherent ‘background’ contains a significant amount of inelastic scattering that is not negligible (Barker & Mildner, 2015 ▸), as usually considered in SANS. Inelastic scattering occurs at high *Q* values and adds to the scattering signal at small angles as a result of multiple scattering. In this case, the incoming monochromatic neutrons are thermalized in the sample and mostly scattered to higher energies than the incoming (monochromatic) energy, hence having shorter wavelengths. The amount of this inelastic scattering that is detected in a SANS experiment depends on the detector efficiency at short wavelengths. Therefore, the effects of this contribution to the measured SANS intensity are instrument specific (Barker & Mildner, 2015 ▸).

Ghosh & Rennie (1990 ▸) and Rennie & Heenan (1993 ▸) have discussed in detail the effect of the inelastic scattering on the normalization of SANS. The effect of the inelastic scattering is particularly critical for SANS instruments that are genuinely designed to work in time-of-flight (TOF) mode, owing to the wide wavelength band used in this case and complications with the wavelength-dependent normalization (Dewhurst, 2008 ▸; Do *et al.*, 2014 ▸; Sokolova *et al.*, 2019 ▸).

Especially in the case of single proteins or single polymer chains in solution, in order to obtain the correct coherent-scattering contribution, the focus is on detection and normalization in SANS, but when subtracting the ‘incoherent background’, the incoherent inelastic scattering should be taken into account. Typically, these are small structures that deliver weak coherent signals at high *Q* values, under the dominating incoherent scattering. The same applies to local details of large structures, especially in the case of monodisperse biomolecules. On the other hand, the nearly flat scattering from the solvent (buffer) predominates at high *Q* in the experimental SANS data, which by far exceeds the total scattering from the solute. Resolving such structures requires more attention in the case of contrast variation conditions, when mixtures of protonated and deuterated solvents are involved. First of all, the incoherent component from the solvent (buffer) sample that is used for background subtraction differs from that of the sample of interest because of the solute. Furthermore, owing to nonlinearities in hydrogen- and deuterium-scattering contributions, extrapolation of some results for H–D mixed conditions cannot be used for other conditions because of the different path lengths for different amounts of hydrogen (H) and deuterium (D) in the solvent (Rubinson *et al.*, 2008 ▸). The measured incoherent scattering is considerably larger than the scattering calculated from the known sample composition (Brûlet *et al.*, 2007 ▸) as a result of multiple scattering effects, which depend on the sample geometry, wavelength and isotopic sample composition (Carsughi *et al.*, 2000 ▸; Shibayama *et al.*, 2005 ▸). The reference coherent and incoherent cross sections (Sears, 1992 ▸) are calculated from the bound scattering length for nuclei and neglect inelastic effects arising from interchange of energy with neutrons. This approximation is reasonable for the incoherent scattering from heavy elements. However, the actual cross sections for the light elements depend on the neutron wavelength and sample temperature, and the reference values are a lower limit. Experiments have shown that the incoherent scattering from hydrogen can be considerably larger than its bound values (Maconnachie, 1984 ▸; Glinka, 2011 ▸). The neutrons change not only energy due to inelastic effects but also momentum. Because the structure factor of liquids *S*(*Q*) is obtained by the integration of the scattering function *S*(*Q*, ω) over the energy transfer ω at constant *Q*, it is necessary to correct the neutron diffraction data for the distortions induced by the dynamical effects, which was first studied in detail by Placzek (1952 ▸). Placzek’s correction could be derived from the ratio of the mass of the neutron to the mass of the scattering atom and the first and second moments of *S*(*Q*, ω), and works well in the case where the mass of the neutron is much smaller than that of the scattering atom (Soper, 2009 ▸). The corrections that should be applied in the case of light atoms, such as hydrogen, deuterium or helium, have been reported by Soper (2009 ▸).

A detailed survey of different background sources in SANS, which contains a thorough discussion on the incoherent background, has been reported more recently by Barker & Mildner (2015 ▸).

Finally, using spin-flipped neutrons and polarization analysis with ^3^He neutron-spin filters, one can unambiguously distinguish coherent nuclear scattering from nuclear spin-incoherent (NSI) scattering, owing to the nonzero probability of neutron spin flip (SF) for spin-incoherent nuclear scattering in contrast with zero probability of SF for coherent nuclear scattering. For hydrogen, two-thirds of NSI single scattering occurs in the SF channel and one-third occurs in the non-spin-flip channel (Gentile *et al.*, 2000 ▸; Chen *et al.*, 2017 ▸). This two-to-one ratio is reduced to nearly one when multiple scattering occurs. Additionally, the polarizing efficiency and transmission are different for inelastically scattered neutrons from the elastic part because of the strong wavelength dependence of the ^3^He analyser. Thus, as a result of this technical and methodical complexity, this method has not yet matured for routine use in SANS on biological and soft-matter solutions. Moreover, the separation of coherent and incoherent scattering in soft-matter and biological samples using this approach is incorrect if it is not accompanied by an appropriate correction to inelastic scattering using TOF (Chen *et al.*, 2017 ▸). The inelastic scattering mixes with the NSI scattering, making it impossible to separate in the SANS polarization analysis method currently in use.

As already stated by Heenan & Rennie (1993 ▸), in order to control the incoherent background due to inelastic multiple scattering, it is useful to equip the pinhole SANS instruments at steady-state neutron sources with an option that operates with a pulsed monochromatic incident beam and TOF data acquisition. The present communication reports the results obtained at the KWS-2 high-intensity SANS diffractometer (Radulescu *et al.*, 2012 ▸) during the commissioning of an option that enables the separation of the inelastic and elastic scattering from the sample of interest. The option involves the use of a compact chopper and TOF data acquisition, and enables data analysis by considering only the elastic contribution. We report here on the testing of different materials used in SANS experiments in different relevant conditions for routine measurements at KWS-2. We also discuss what one can learn at KWS-2 in a quick manner, during the experiment, about the incoherent inelastic background from soft-matter and biological samples, and how one can collect data with reduced incoherent background, on demand. This new option will pave the way for a more accurate detection of weak scattering signals from small structures, *e.g.* from small proteins or short polymer chains in solution, when combined with the wide-angle detection option, for reaching *Q*_max_ = 2 Å^−1^, and with the polarization-analysis option using spin-exchange optical pumping (SEOP) polarized ^3^He neutron-spin filters (Salhi *et al.*, 2017 ▸), which are currently in development at KWS-2.

## 2. Experimental details   

A secondary single-disc compact chopper was designed and optimized to pulse the incoming monochromatic beam and enable TOF measurement of inelastically and elastically scattered neutrons under the typical high-*Q* conditions at the KWS-2 SANS diffractometer, *i.e.* a short wavelength of incoming neutrons λ_i_ and short sample-to-detector distance *L*_D_. Unlike the main double-disc chopper of the instrument (Radulescu *et al.*, 2015 ▸), which is installed at a fixed position in front of the collimation system and is used to improve the wavelength resolution over the entire *Q* range, the secondary chopper operates at the sample position. With a weight of only 36 kg, including the splinter protection box and the installation frame, the new chopper is portable and can be quickly and precisely installed at a predefined position, in front of the sample stage, on demand, without requiring technical assistance for positioning or a calibration check. The 8% duty-cycle chopper disc has four windows with a fixed opening of 1 cm and can operate in air at selected frequencies within the range *f* = 15–50 Hz, to match the TOF conditions for a given λ_i_ within the range 4.5–7 Å and for *L*_D_ between 1–4 m. The TOF data acquisition is triggered for each window, and thus four pulses are produced for each disc rotation. The control of the chopper and TOF data acquisition are implemented in the measurement definition and control software of the instrument.

TOF measurements of the scattering from different materials commonly used in SANS experiments at KWS-2, *i.e.* standard samples [Plexiglas, glassy carbon (GC), silver behenate AgBeh], sample containers (quartz) and solvents (H_2_O, D_2_O), were carried out with λ_i_ = 5 and 7 Å at *L*_D_ = 1.1 and 1.6 m. The H_2_O and D_2_O (99.9% isotopic purity, Sigma–Aldrich) solvents were investigated at different temperatures *T* and for different sample thicknesses *t*_s_ and beam sizes on the sample. A collimation length of *L*_c_ = 4 m and an opening of the entrance aperture of 50 × 50 mm were always used. Thus, the highest flux condition at KWS-2, which can be obtained with a collimation length of *L*_c_ = 2 m (Radulescu *et al.*, 2016 ▸), was not used in this investigation.

The absolute calibration standard at KWS-2 is a slab of Plexiglas with *t*_s_ = 1.5 mm. The GC is a plate with *t*_s_ = 1 mm and was characterized in advance with small-angle X-ray scattering (SAXS) (Zhang *et al.*, 2010 ▸). The AgBeh is a powder sample that is contained in a tightly sealed quartz cuvette with *t*_s_ = 1 mm. As a proof of principle for the data-analysis method, considering only the elastic region of the TOF spectrum for an improvement in the background correction, we prepared a protein solution of 18.12 mg ml^−1^ bovine serum albumin (BSA, Sigma–Aldrich) in D_2_O, without buffer or added salt. To ensure there were no aggregates present in the solution, it was successively filtered using syringe filters of 0.2 and 0.02 µm (Whatman, Anotop). The pH of the solution was 6.8 (Methrom), above the isoelectric point of BSA at 4.7 (Carter & Ho, 1994 ▸), suggesting that the protein was in a globular state but mostly negatively charged. The final concentration was determined using UV/Vis spectrometry (Cary 300) and 0.1 mm quartz cuvettes (Hellma, Germany), on the basis of the extinction coefficient ε_280nm_ = 49 915 *M* ^−1^cm^−1^. Liquid samples were measured in rectangular quartz cells (Hellma, Germany). Measurements on H_2_O were carried out with *t*_s_ = 0.5, 1 and 2 mm, while on D_2_O they were carried out with *t*_s_ = 1, 2 and 5 mm, with the variation of the beam size on the sample between 5 × 5 mm and 8 × 8 mm and a sample temperature between 298 and 343 K. The protein in the D_2_O solution was measured for *t*_s_ = 2 and 5 mm at *T* = 298 K with a beam size of 8 × 8 mm on the sample. The measurement time for different samples was as follows: 600 s for Plexiglas, GC and AgBeh; 600 or 900 s for H_2_O; 900 or 1200 s for D_2_O; and 1200 s for the solution of BSA in D_2_O. The data analysed over the whole TOF spectrum or only in the elastic region were normalized to the upstream monitor, corrected on a pixel-to-pixel basis for the empty cuvette contribution and instrument background following the typical SANS reduction procedure (Radulescu *et al.*, 2016 ▸), and radially averaged to deliver one-dimensional data. Calibration in absolute units (cm^−1^) was carried out using either the Plexiglas and GC standards, in the case where the full TOF spectrum was considered, or the GC standard, when only the elastic region was analysed. The absolute calibration using Plexiglas was applied on corrected two-dimensional data sets before the data were radially averaged, while the calibration with the GC standard was applied on one-dimensionally averaged data using the known scattering cross section of the standard sample as provided by the SAXS calibration procedure (Zhang *et al.*, 2010 ▸) and cross checked with neutrons against the vanadium primary standard (Houston *et al.*, 2018 ▸). The correction for the solvent contribution in the case of the data from the protein solution was applied on one-dimensional corrected and calibrated data.

## 3. Results and discussion   

**Figure 1:**
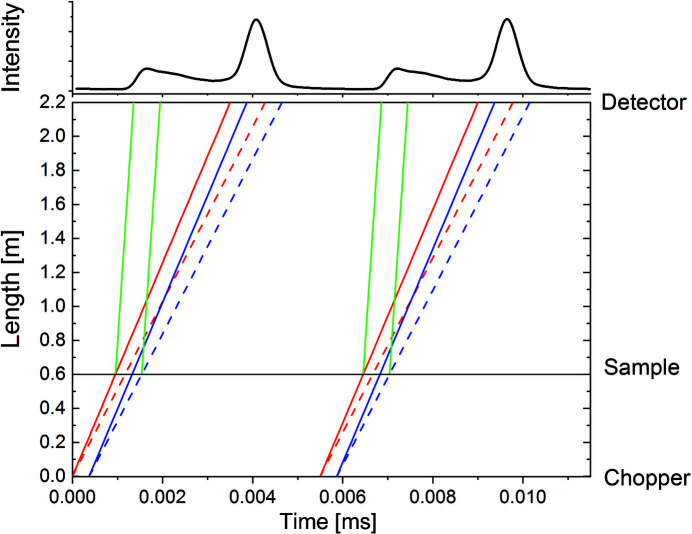
A time–distance diagram for the new chopper of KWS-2 for *L*_D_ = 1.6 m and λ_i_ = 7 Å (bottom), and the neutron intensity recorded on the detector in TOF mode for two bursts produced by the chopper (top). The path of the elastically scattered neutrons (red and blue lines) is shown along with that of the inelastically scattered neutrons (green line) for the sample with λ_s_ = 1 Å. The meaning of different line types and colours is discussed within the main text.

Fig. 1 ▸ shows an example of a time–distance diagram that presents the effect of the secondary chopper on the detection of the neutrons scattered by a 1 mm thick H_2_O sample. The chopper transforms the continuous incoming beam into a pulsed beam, emitting four pulses for each rotation. The pulsed beam hits the sample which is placed at a short distance after the chopper, *L*_CS_ = 0.6 m. Given the current geometry at the sample position of KWS-2, *L*_CS_ must be varied to a certain extent to avoid shadows on the detector margins, depending on the targeted *L*_D_ and hence on the *Q* range. The design and construction of wide-angle detector banks at KWS-2 is currently in progress, taking the TAIKAN BL-15 SANS instrument installed at J-PARC (Takata *et al.*, 2015 ▸) as a model. This option will enable the detection of neutrons scattered up to an angle of θ_s_ = 50°. This will imply modification of the entrance geometry of the detector tank and future wide-angle detector wings. The resulting increased flexibility in the sample positioning will enable one to avoid shadows on the detector for the shortest *L*_CS_ ≃ 0.02 m, which is dictated by the splinter shield of the chopper.

In the current experiment, the elastically and inelastically scattered neutrons from the sample were recorded on the detector as a function of the flight time. Following the TOF neutron scattering principles (Copley, 1990 ▸), a good separation of the elastic and inelastic scattering from the sample could be achieved. The elastic line includes the broadening due to the wavelength distribution provided by the velocity selector (Δλ/λ = 10%), with the continuous and dotted lines indicating the fastest and slowest neutrons. The red and blue lines indicate the width of the neutron pulse provided by the chopper opening, which spreads in time on the way to the detector, due to Δλ/λ. Finally, the trajectories of the inelastically scattered neutrons with λ_s_ = 1 Å due to thermalization in the sample are shown in green.

**Figure 2:**
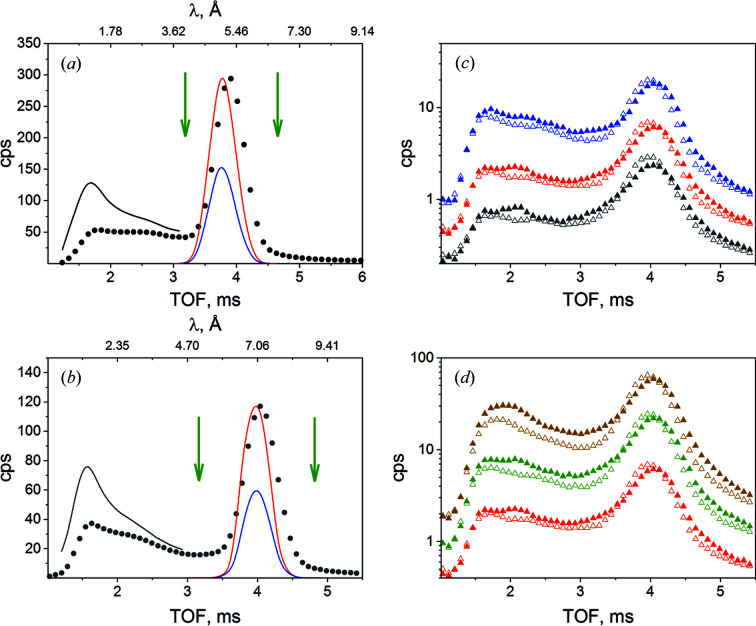
(*a*), (*b*) TOF scattered and transmitted spectra from a 1 mm H_2_O sample at 298 K measured with incident-beam wavelengths of λ_i_ = 5 Å (*a*) and 7 Å (*b*) at *L*_D_ = 1.6 m. (*c*), (*d*) TOF scattered spectra from D_2_O samples at 298 K measured with an incident-beam wavelength of λ_i_ = 7 Å for samples with different thicknesses with a beam size of 5 × 5 mm (*c*), or for a sample thickness of *t*_s_ = 2 mm with different beam sizes (*d*). The meaning of different line types and colours is discussed within the main text. The vertical arrows in (*a*) and (*b*) indicate the elastic part of the spectrum used in the data analysis.

Using the list-mode data acquisition which enables time-stamped measurements (Houston *et al.*, 2018 ▸), the pulses are summed together at the end of the measurement and one single TOF file is produced. Currently, 64 TOF channels with an adjustable width to match the TOF conditions are used in the acquisition option. The data can be further processed for the entire or for selected TOF ranges. TOF spectra of the scattered and transmitted intensities from an H_2_O sample (*t*_s_ = 1 mm), which were acquired for different λ_i_ at the same *L*_D_, are shown in Figs. 2 ▸(*a*) and 2 ▸(*b*) (symbols). The transmitted intensities were recorded in the centre of the detector, also in TOF mode, after they had passed through the central window of the beam stop, which is covered with an attenuating boronated glass. The transmitted intensities from the water in the quartz cuvette (blue line) and from the empty cuvette (red line) are also shown. Knowing the parameters λ_i_, *L*_D_, *L*_CS_ and the TOF, we can calculate the wavelength of the neutrons arriving on the detector (top axes of the plots), assuming that all neutrons are detected at the same *L*_D_, as given by the centre of the detector. As will be discussed in detail below, this is a crude assumption for short detection distances, where the trajectories of the neutrons are slightly longer than *L*_D_, depending on the distance of the detection point from the centre of the detector. On the basis of the estimated wavelength of the collected neutrons in TOF mode, the corrected intensity for the detection efficiency of the ^3^He detector for different λ in the inelastic region of the spectrum was obtained [black lines in Figs. 2 ▸(*a*) and 2 ▸(*b*)]. For a better comparison of the inelastic effects in H_2_O and D_2_O, Fig. S1 of the supporting information shows on the same plot the TOF spectra for different sample thicknesses *t*_s_, normalized to the measurement time. The inelastic contribution from the 0.5 mm thick H_2_O sample is comparable to that from the 5 mm thick D_2_O sample. Considering also the isotopic purity of the heavy water used in the experiment (99.9%), we may assign the inelastic contribution from the D_2_O samples as arising from the D nuclei (Barker & Mildner, 2015 ▸).

The effect of the neutron trajectories on their TOF can be observed in the slight shift toward shorter TOF for the transmitted beam that is collected in the middle of the detector compared with the elastic peak of the TOF spectrum that is collected over the entire detector and is thus averaged over a wider angular range. The effect of detection angle on the TOF spectrum may be clearly observed in Figs. 2 ▸(*c*) and 2 ▸(*d*), which present the scattered intensities from D_2_O samples at 298 K measured with the incident-beam wavelength λ_i_ = 7 Å with a beam size of 5 × 5 mm for sample thicknesses *t*_s_ = 1 mm (black), 2 mm (red) and 5 mm (blue) [Fig. 2 ▸(*c*)], or for sample thickness *t*_s_ = 2 mm with different beam sizes on the sample (5 × 5 mm – red, 6 × 8 mm – green, and 8 × 8 mm – brown) [Fig. 2 ▸(*d*)] . In Figs. 2 ▸(*c*) and 2 ▸(*d*) the filled symbols represent the data collected at the rim of the detector (high scattering angles) while the open symbols show the data collected on the central part of the detector (small scattering angles), always on sectors of comparable detection area. Inelastic scattering is a *Q*-dependent process (Arbe *et al.*, 2020 ▸). Therefore, the effect of the inelastic and quasi-elastic scattering processes can be better observed in the TOF spectra measured at higher scattering angles [full symbols in Figs. 2 ▸(*c*) and 2 ▸(*d*)]: the peaks at 1 and 2 ms flight times in the inelastic spectrum, and the broadening at the base of the elastic line due to quasi-elastically scattered neutrons with flight times between 3 and 5.5 ms. Unlike this, the spectra collected at small scattering angles are mainly a consequence of multiple scattering processes. Therefore, only the short-flight-time feature, with short wavelength or high energy, is clearly recognizable in the inelastic spectrum. Also, because of the shorter trajectories in this case, the elastically scattered neutrons are characterized by shorter times, as in the case of those collected at the rim of the detector. The multiple scattering effects become stronger with the increase in *t*_s_ or beam size on the sample (Carsughi *et al.*, 2000 ▸; Shibayama *et al.*, 2005 ▸; Brûlet *et al.*, 2007 ▸), which is why the inelastic spectra collected at higher scattering angles for *t*_s_ = 5 mm or for a beam size on the sample of 8 × 8 mm seem to have fewer features than the corresponding spectra from thinner samples or measured with smaller beam sizes. However, we are not interested in implementing a genuine TOF working mode at KWS-2. Therefore, the newly implemented option represents a sufficient way to understand the effect of the inelastic scattering on the quality of data that are measured at a pinhole SANS instrument and provides a possibility to treat only the elastically scattered neutrons from the sample of interest and learn about improvement in background correction.

**Figure 3:**
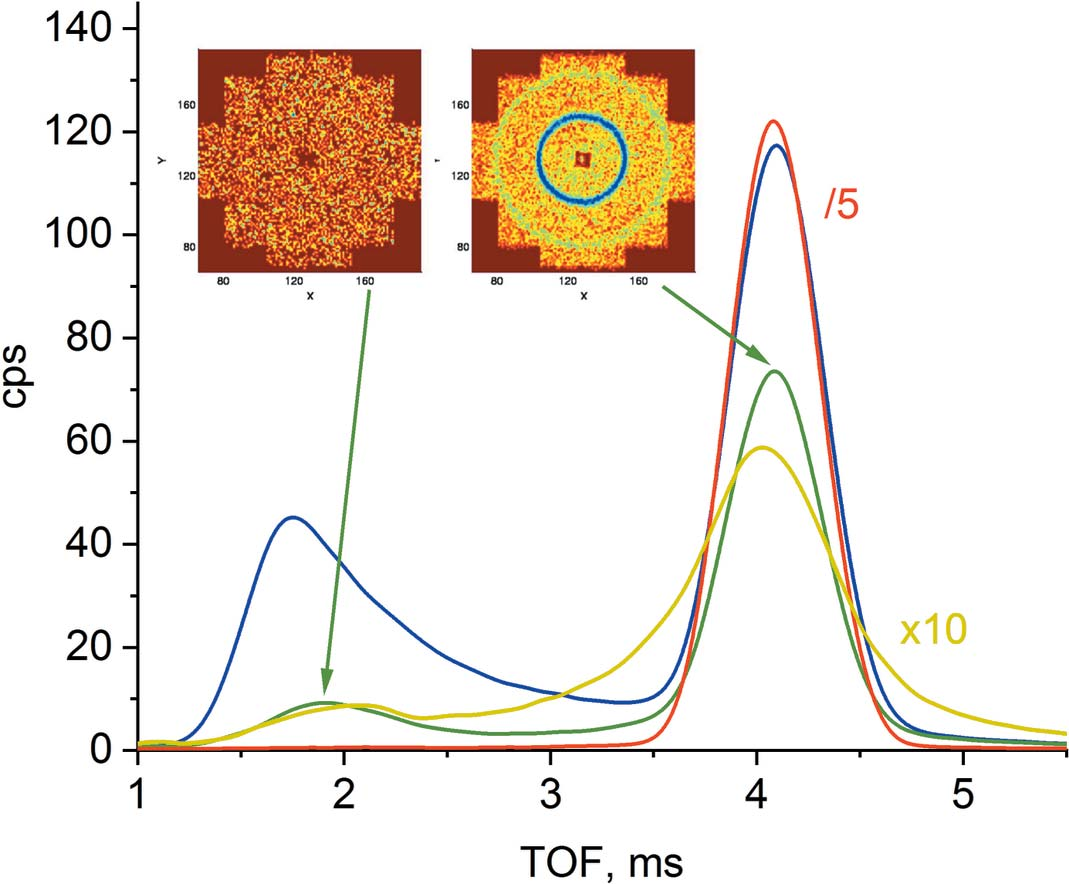
TOF spectra recorded at *L*_D_ = 1.6 m with λ_i_ = 7 Å from AgBeh (green), empty quartz cell (dark yellow), GC (red) and Plexiglas (blue). The two-dimensional scattering patterns are collected within the elastic (right) and inelastic (left) regions of the TOF spectrum from AgBeh.

The TOF spectra from different materials used in the experimental routine at KWS-2 are shown in Fig. 3 ▸ as collected at *L*_D_ = 1.6 m with λ_i_ = 7 Å. The Plexiglas standard sample yields a strong inelastic scattering that can be observed at short flight times. On the other hand, the GC scatters almost completely elastically. Thus, the GC may be used for the calibration of the scattering from other samples when only the elastic spectrum is considered for the data analysis. The empty cuvette (quartz, *t*_s_ = 2.5 mm) produces weak scattering, with a very weak inelastic contribution. AgBeh clearly delivers inelastically scattered neutrons, which contribute to the incoherent background, since it does not contain structural information, as shown by the two-dimensional scattering pattern observed at short flight times. The structural information is found in the coherent scattering, which is observed as rings in the two-dimensional scattering patterns within the elastic region of the spectrum.

**Figure 4:**
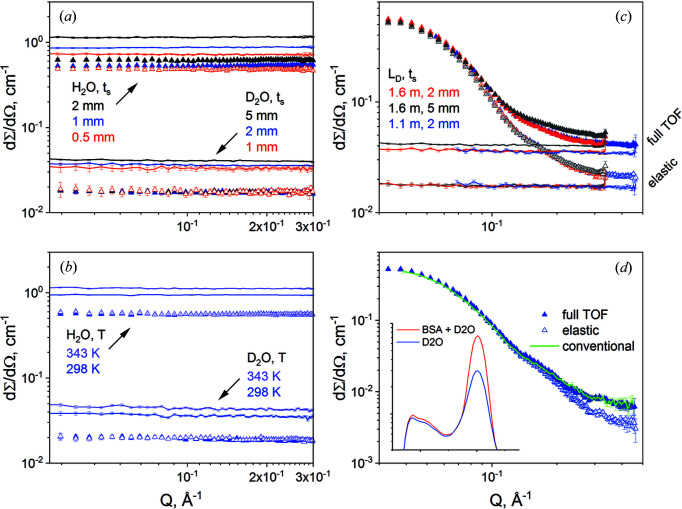
Scattering cross sections in absolute units obtained from the reduction of data collected in TOF mode: H_2_O and D_2_O samples with different thicknesses *t*_s_ at 298 K (*a*) and with a thickness of *t*_s_ = 1 mm at different temperatures (*b*); BSA solution in D_2_O (symbols), together with the D_2_O solvent alone (lines) (*c*); and BSA in solution after the correction for the D_2_O contribution (*d*). The lines in (*a*) and (*b*) are results obtained from the data collected over the full TOF spectrum, while the symbols correspond to data analysed only in the elastic region. The results in (*c*), obtained by reducing the data collected at different *L*_D_ for different *t*_s_ considering either the full TOF spectrum or only the elastic region, are differentiated by colours or lateral indications. The data in (*d*) are shown together with the result of a conventional SANS measurement (green line), while the inset in (*d*) shows the TOF spectra from the BSA solution in D_2_O and D_2_O alone.

Data in absolute units from H_2_O and D_2_O for different sample thicknesses *t*_s_ and temperatures *T* are presented in Figs. 4 ▸(*a*) and 4 ▸(*b*). The data collected over both the entire TOF spectrum (lines) and only the elastic region (symbols) are displayed.

This analysis shows that the inelastic fraction that is contained in the data collected over the full TOF spectrum increases with increasing sample thickness or temperature on the sample. One possible explanation is the increase in the multiple scattering events with the increase in the flight path of neutrons through the sample or the water temperature governing the thermalization of neutrons. The effect of the inelastic fraction is striking in the case of D_2_O when the scattering cross section of the elastic scattering represents about half of that measured over the entire TOF spectrum. For the H_2_O sample of *t*_s_ = 1 mm the elastic scattering cross section is ∼0.6 cm^−1^, a value close to that reported by Heenan (2015 ▸). The elastic scattering from the H_2_O sample at 298 K is also affected by strong multiple scattering effects, as indicated by the variation of the elastic data with *t*_s_. For all other cases, the elastic data lie on top of each other, which indicates a constant contribution of the multiple scattering to these spectra. We may thus conclude that for H_2_O and D_2_O, which are solvents commonly used in SANS investigations of soft-matter and biological systems, the variation of both sample thickness and temperature cause variation in the inelastic scattering, and hence variation in the collected background in usual SANS experiments. The plots in Fig. S2 show the same results for H_2_O and D_2_O with a linear vertical axis instead of a logarithmic one, which makes more obvious the magnitude of the decrease when only the elastic part of the spectrum is taken into account and enables a better assessment of how flat the backgrounds are. Our experimental values shown in Figs. 4 ▸(*a*) and 4 ▸(*b*) are similar to those reported by Shibayama *et al.* (2009 ▸). They proposed a method to evaluate (dΣ/dΩ)_inc_ for hydrogenous samples without using the values for scattering lengths/cross sections provided by Sears (1992 ▸) or reference samples, which was called ‘the transmission method’. The ‘*T* method’ uses the transmission *T*_s_ and the thickness *t*_s_ of the sample, and since both *T*_s_ and *t*_s_ are values routinely obtained in a SANS measurement for data reduction, the ‘*T* method’ is quite useful. The applicability of this method was examined on strong incoherent scattering samples such as H_2_O and D_2_O with the sample thickness between 1 and 4 mm, and it was found to be successful (Shibayama *et al.*, 2009 ▸). A detailed analysis of our data on H_2_O, D_2_O and mixtures of H_2_O/D_2_O (not shown here) in terms of this method is in progress and will be reported elsewhere.

The scattering patterns from the solution of BSA in D_2_O are shown in Fig. 4 ▸(*c*) alongside those from the D_2_O solvent alone, as they were measured for different *t*_s_ at different *L*_D_ and reduced according to the usual SANS procedure (Radulescu *et al.*, 2016 ▸). The measured data were reduced by considering either the full TOF spectrum (the upper curves) or only the elastic region of it (the lower curves) and calibrated in absolute units using the GC as the standard. The absolute calibration of the data analysed over the entire TOF spectrum using the Plexiglas standard produced similar results to the case where these data were calibrated using the GC. Similar to the case of H_2_O and D_2_O (Fig. 2 ▸), the inelastic component of the TOF spectrum from the protein solution yields an additional background that is observed in the high-*Q* region, which increases with the thickness of the sample. The reduction and calibration procedures include normalization to sample thickness so that the observed change in background is due to multiple scattering.

According to Nagy *et al.* (2016 ▸), a correlation peak appears in the *Q* region 0.05–0.1 Å^−1^ of the scattering pattern of BSA solutions in D_2_O at concentrations above ∼10 mg ml^−1^ because of the intermolecular interactions. Being interested in the effect of the inelastic scattering on the background, we focused only on measurements above the *Q* value of the correlation maximum. An interesting feature of the highly structured BSA (due to a high content of α helices) is highlighted in the high-*Q* region: the shoulder at 0.15 Å^−1^ arising from the twofold structure of the BSA monomer (Ameseder *et al.*, 2019 ▸). When only the elastic region of the TOF spectrum is considered for data analysis, after the correction for the solvent scattering is applied, a lower background level is obtained at high *Q* compared with the case where the whole spectrum is analysed [Fig. 4 ▸(*d*)]. The inelastic scattering observed in the TOF spectra is mainly due to D_2_O. However, the inelastic intensity from the protein solution lies clearly above the D_2_O intensity [inset of Fig. 4 ▸(*d*)], which is a consequence of the inelastic scattering from the protein and the additional protons in D_2_O due to the H/D exchange in BSA. The result of the conventional measurement (the green line in Fig. 4 ▸) coincides with the scattering pattern obtained from the TOF measurement when the whole TOF spectrum was analysed, hence considering all scattered neutrons as in a usual SANS experiment. The elastic data [open symbols in Fig. 4 ▸(*d*)] are still affected by some incoherent background from the BSA, which explains the still increased scattering level towards high *Q*. In order to be able to better assess the effect of discarding the inelastic data in the data reduction and evaluation, the scattering profile of the BSA from the atomic coordinates (PDB ID 4f5s; Bujacz, 2012 ▸) was estimated using the software *CRYSON* (Svergun *et al.*, 1998 ▸). The experimental results in the high-*Q* range are shown together with the calculated curve in Fig. S3.

There is always a trade-off between the signal-to-noise improvement, based on the beam pulsing and TOF analysis, and the counting statistics (Barker & Mildner, 2015 ▸). However, at high-intensity SANS diffractometers like KWS-2 (Radulescu *et al.*, 2012 ▸), it can be affordable to use this method for gaining knowledge about the inelastic scattering and the background that occurs because of it, which is useful if the data at high *Q* do not show a flat profile. In the current experiments, owing to the use of the chopper we were working with an intensity on the sample representing *ca* 8% of the nominal intensity provided by the monochromatization/collimation setup. This situation may be improved to some extent by a further optimization of the chopper disc. The improvement in background correction will be very useful in the near future, when the wide-angle detection option will allow us to cover a *Q* range up to *Q*_max_ = 2 Å^−1^ while having large area detectors installed in a fixed position at *L*_D_ ≃ 1.5 m, a setup that will enable optimal TOF measurement conditions. Small proteins in solution yield form-factor scattering details in the *Q* region from 0.5 to 1.5 Å^−1^ (Hirai *et al.*, 2019 ▸). The improvement based on the separation of the inelastic scattering and the data analysis carried out only on the quasi-elastic data via TOF data acquisition may help in reaching a better observation and characterization of such weak scattering details. Moreover, the polarization analysis, which is also in development at KWS-2, will benefit from the possibility to correctly separate the inelastically and elastically scattered neutrons from hydrogen-containing samples.

## 4. Conclusions   

The neutrons scattered inelastically on hydro­carbon materials such as polymer or protein solutions provide a significant contribution to the recorded intensity in a SANS experiment. This contribution is due to incoherent and multiple scattering on hydrogen or deuterium nuclei and adds up as a flat background to the measured data. At the high-intensity pinhole SANS diffractometer KWS-2 we installed a new chopper just in front of the sample stage, which enables one to pulse the beam and record the neutrons in TOF mode. A good separation of the inelastic and elastic scattering from different materials that are routinely used in experiments at KWS-2, such as standard samples, sample containers and common solvents under different temperature and sample-thickness conditions, was obtained while working in the typical high-*Q* setup at KWS-2, namely short incoming wavelength, λ_i_ = 5– 7 Å, and short detection distances, *L*_D_ = 1.1–1.6 m. As observed in a proof-of-principle experiment on samples of BSA solution in D_2_O with different thicknesses, a lower background is obtained than with the usual measurement method if only the elastic contribution in the recorded TOF results is considered. This option can be optimally used in the near future at KWS-2 when the newly planned options (namely the wide-angle measurement mode and spin-flipped neutrons together with polarization analysis using SEOP ^3^He neutron-spin filters, accompanied by appropriate correction to inelastic scattering using TOF mode) become operational.

## Supplementary Material

Figs. S1, S2 and S3, which help for assessing the data quality and the effect of the proposed method. DOI: 10.1107/S1600576721006610/jl5020sup1.pdf
