# Supplementary material for: Separation of the inelastic and elastic scattering in time-of-flight mode on the pinhole small-angle neutron scattering diffractometer K­WS-2
Source: J Appl Crystallogr. 2021 Jul 30;54(Pt 4):1217–24. doi: 10.1107/S1600576721006610 (PMC8366428; doi:10.1107/S1600576721006610)
Supplement: Supplementary file 1 [file j-54-01217-sup1.pdf]

JOURNAL OF
APPLIED
CRYSTALLOGRAPHY

Volume 54 (2021)

Supporting information for article:

Separation of the inelastic and elastic scattering in time-of-flight mode on the pinhole SANS diffractometer KWS-2

Livia Balacescu, Georg Brandl and Aurel Radulescu

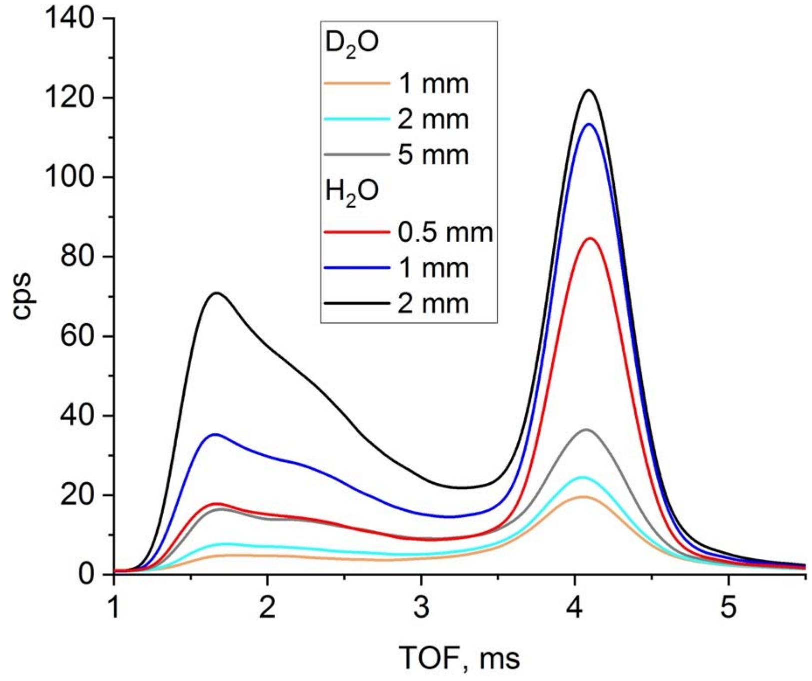

Figure S1 TOF spectra from D_2O and H_2O samples for different sample thickness t_s.

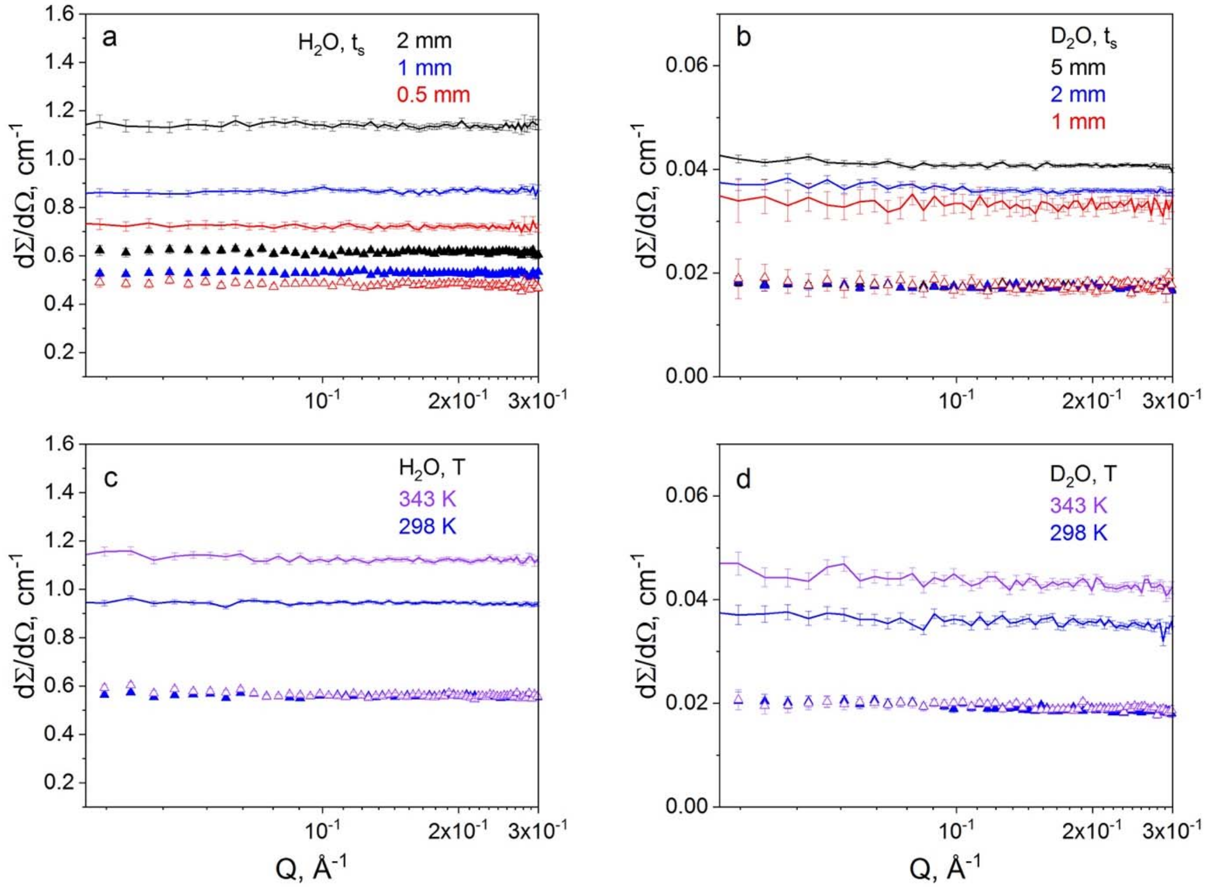

Figure S2 The data from Figure 4a,b, presented on a linear vertical axis instead of a logarithmic one.

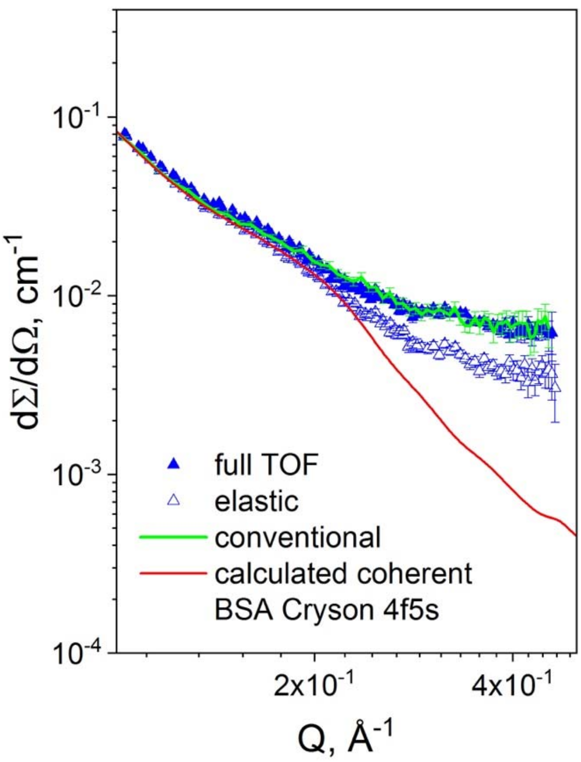

Figure S3 Measured data from BSA in D2O solution (as in Figure 4d) together with the calculated scattering profile with CRYSON software from the atomic coordinates (PDB ID: 4f5s).
